# Supplementary material for: Assembly intermediates of orthoreovirus captured in the cell
Source: Nat Commun. 2020 Sep 7;11:4445. doi: 10.1038/s41467-020-18243-9 (PMC7477198; doi:10.1038/s41467-020-18243-9)
Supplement: Supplementary file 1 — Supplementary Information [file 41467_2020_18243_MOESM1_ESM.pdf]

Assembly intermediates of orthoreovirus captured in the cell

Sutton et al.

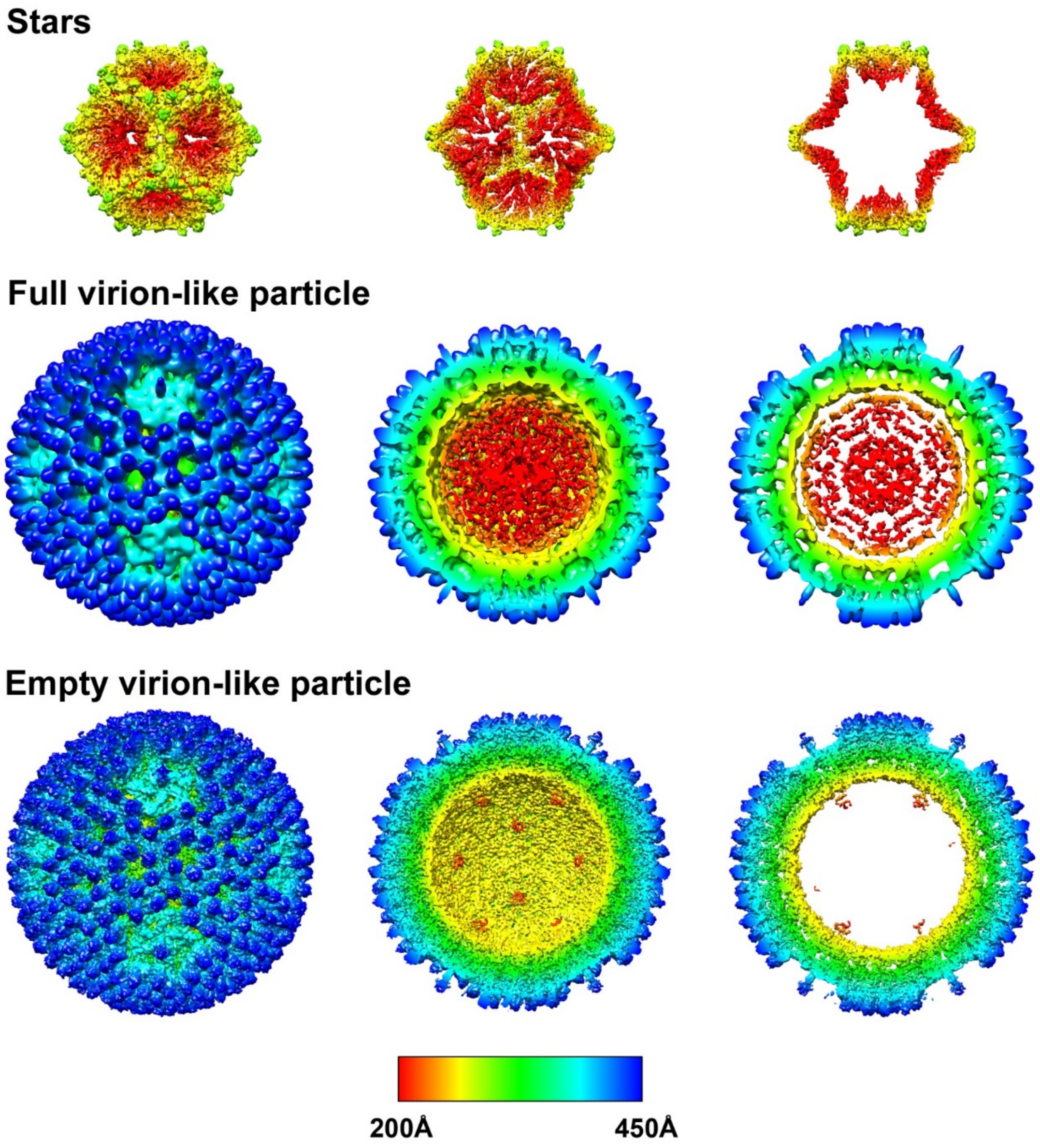

Supplementary Figure 1. Maps coloured by radius for the 3 categories of particles, full and empty virion-like and stars (see Methods). The left hand panels show the whole particles, middle panels the particles are cropped at their centres in the z-direction, and the right hand panels show a thin slice at z=½. Note the density for the polymerase molecules in red in the empty virion-like particles.

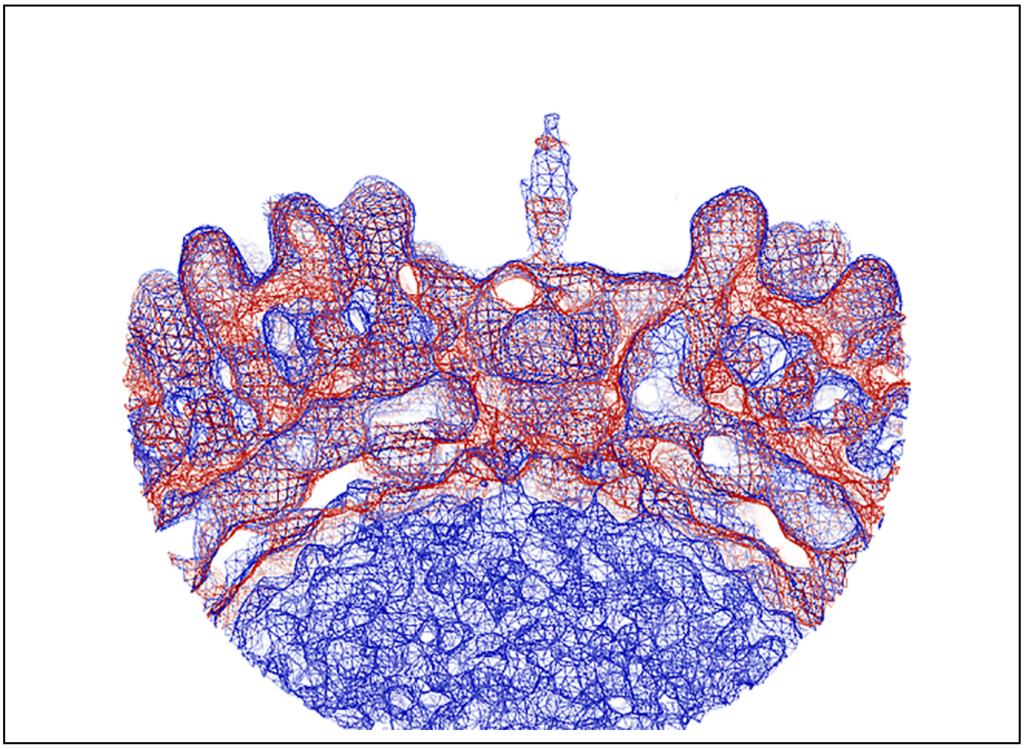

Supplementary Figure 2. Sub-tomogram averaged maps from PEET for full (blue) and empty (red) virion-like particles. Note the density within the full particles (lower portion) and the common 5-fold spikes (centre top).

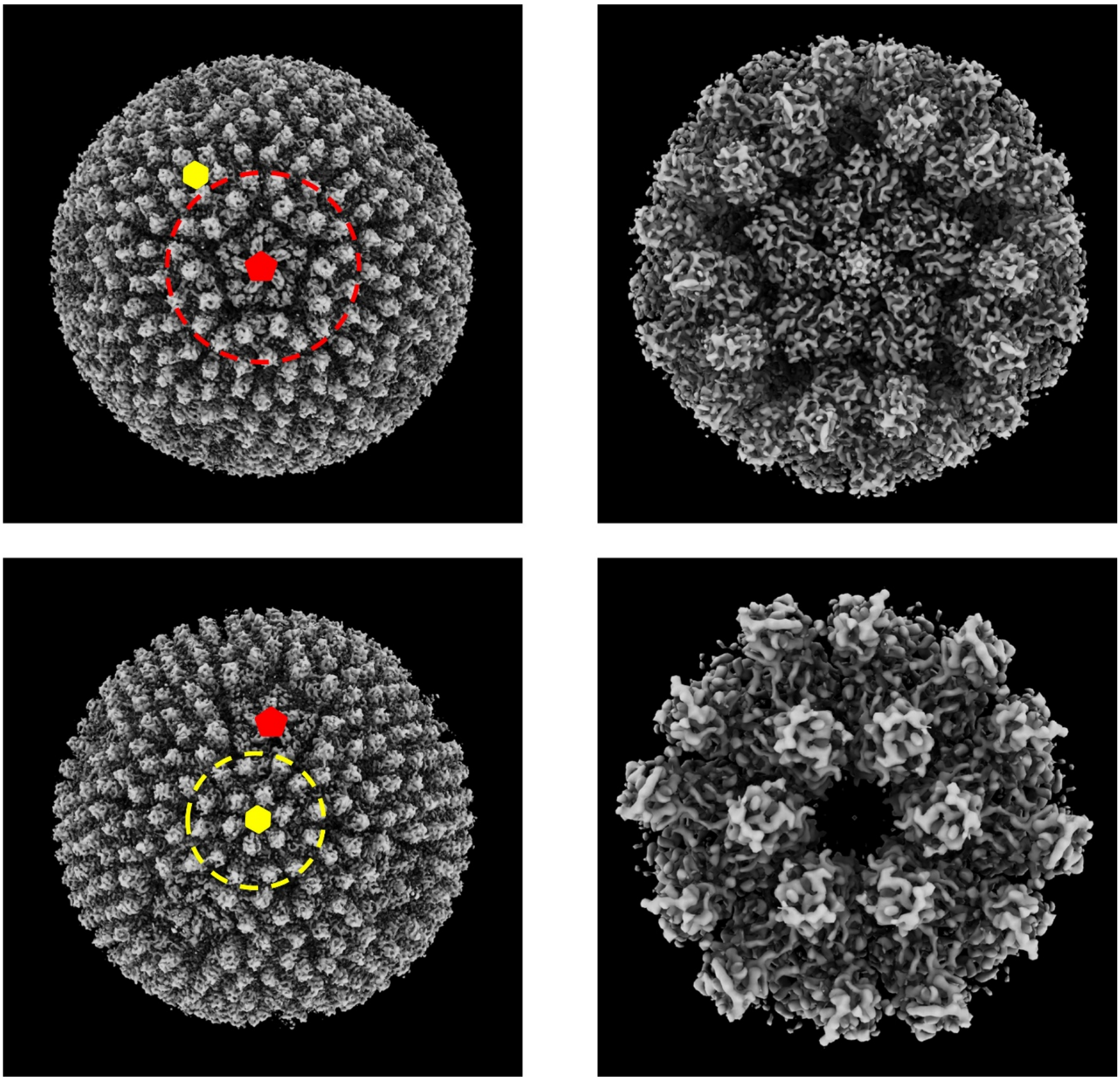

Supplementary Figure 3. Definition of the axes used for local reconstructions in emClarity. Yellow shows pseudo 6-fold, red 5-fold.

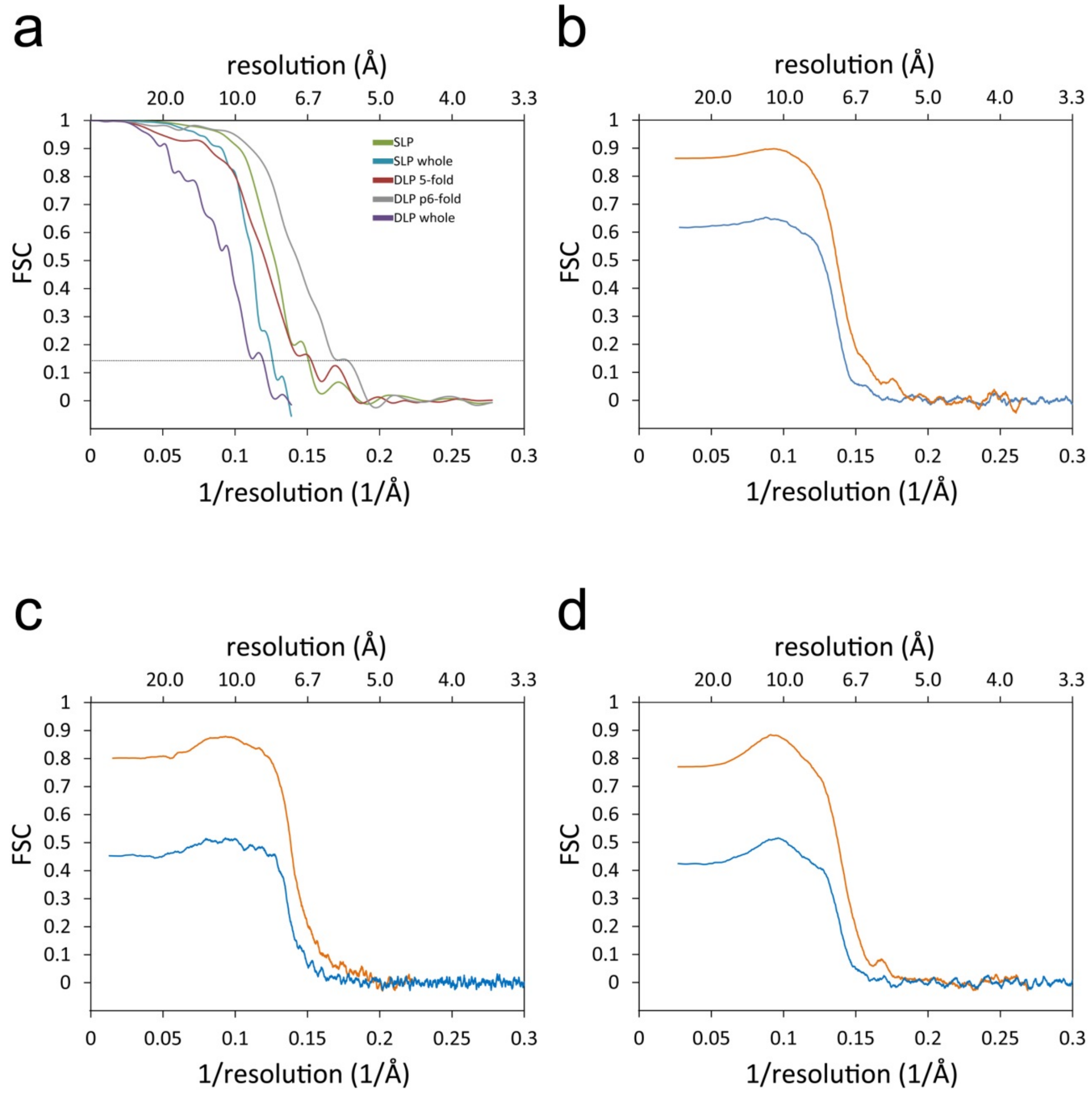

Supplementary Figure 4. **a,** FSC curves for the five sub-icosahedral reconstructions. The FSC is between two half data sets. p6-fold corresponds to the pseudo 6-fold map. **b-d** Representative FSC curves for the fit of the models to the map (as calculated by the Phenix tool phenix.validation_cryoem[1,2]) for **b,** virion-like particle pseudo 6-fold, **c,** virion-like particle 5-fold and **d,** SLP. Masked data is coloured orange, unmasked blue.

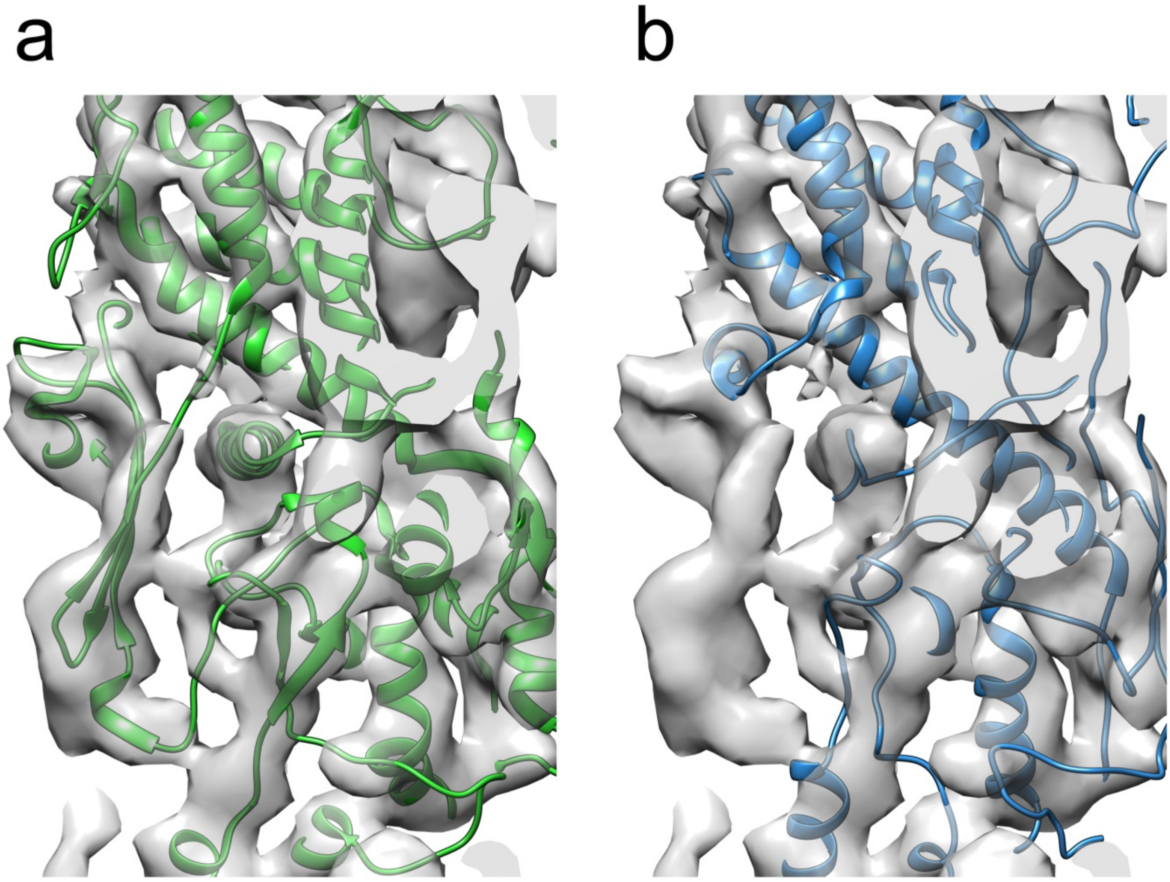

Supplementary Figure 5. Rigid body fits for molecule A of **a,** reovirus λ1 and **b,** rotavirus VP2 into the SLP map. As can be seen there exists substantial density which cannot be accounted for by the rotavirus VP2. However, reovirus λ1 has insertions at residues 843-851 and 997-1014 which occupy this density.

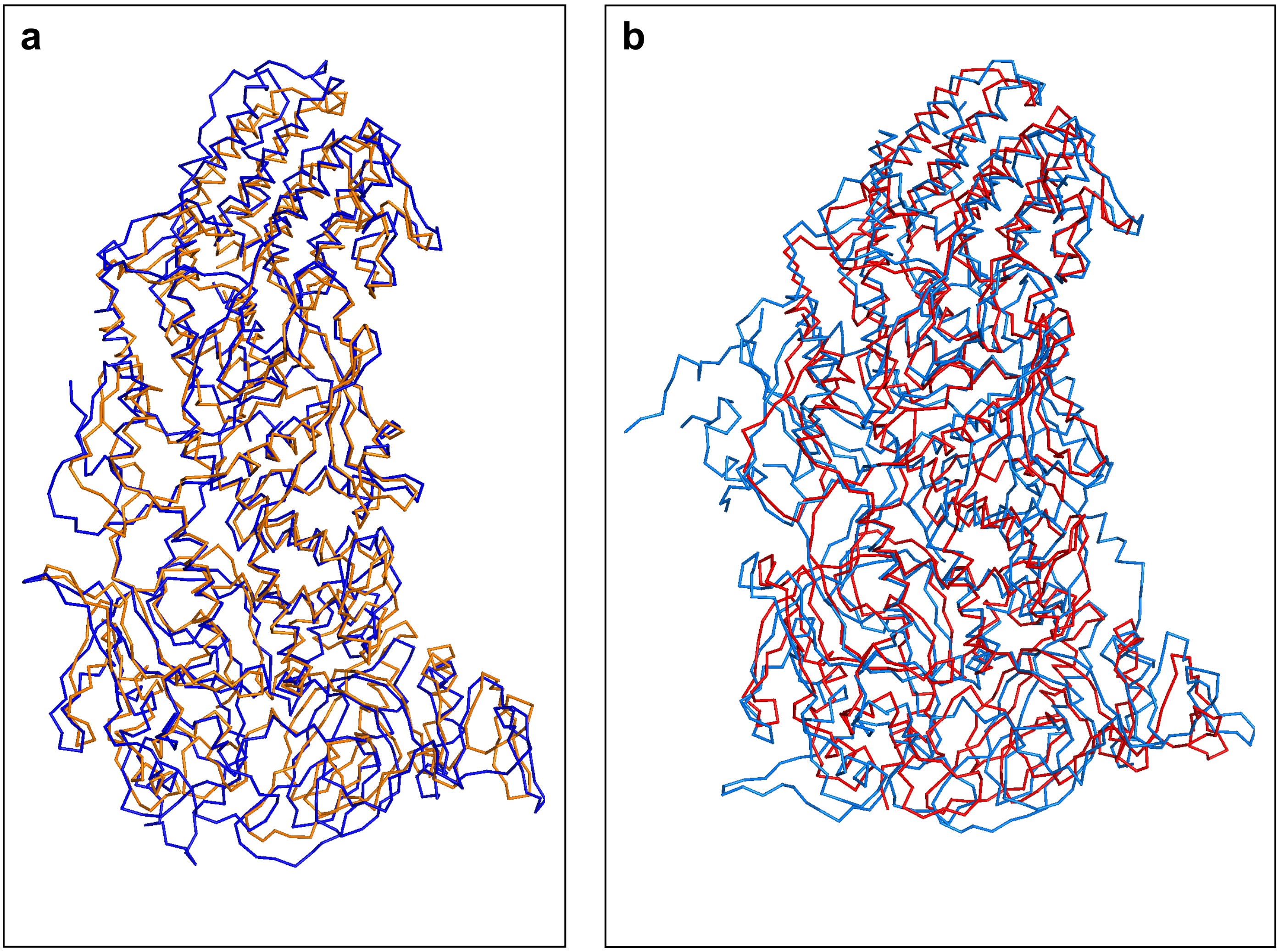

Supplementary Figure 6. Superposition of λ1 molecules from virion-like particles and SLP. a, molecule A with virion-like particle coloured blue and SLP orange. **b,** molecule B coloured pale blue and red for virion-like particle and SLP, respectively.

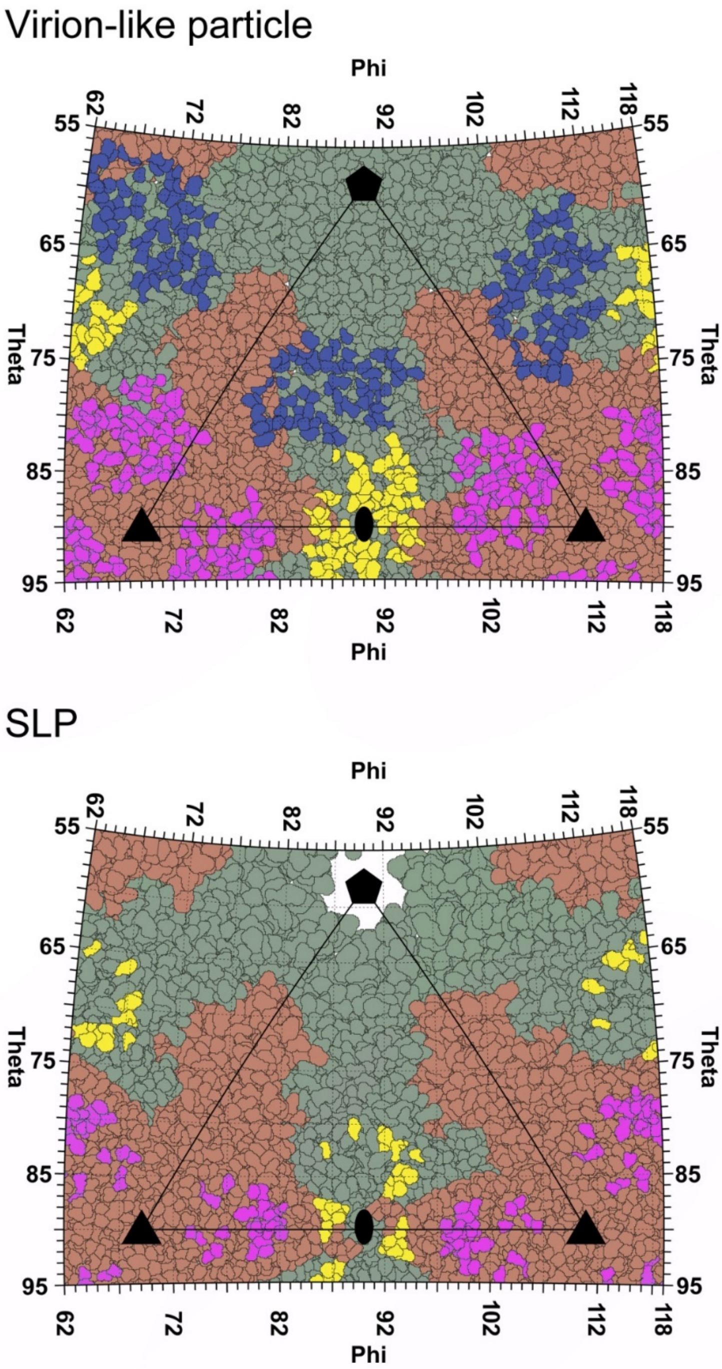

Supplementary Figure 7. Roadmap figure showing the relative positions of the footprints for σ2 on the virion-like particle and the SLP. The λ1 molecules A and B are coloured faded green and red respectively. The footprint for the σ2 A-hinge is coloured blue, σ2 three-fold adjacent is purple and σ2 two-fold yellow. The roadmaps were produced using RIVEM[3].

**Data Acquisition**	Microscope	Krios				
	Voltage	300 keV				
	Detector	K2 Summit				
	Energy filter	Yes, 20 eV slit				
	Number of tomograms	5				
	Defocus range	4.3 to 5.9				
	Acquisition scheme	Dose-symmetric				
	Total dose	82 $e^-/Å^2$				
	Tilt range	-40° to +40° in 2° increments				
**Data Processing**	Map					
	SLP-whole empty*	SLP 5-fold**	DLP-whole empty*	DLP 5-fold**	DLP pseudo 6-fold**	DLP- whole full*
Å/pixel	3.6	1.8	3.6	1.8	1.8	3.6
Number of sub-tomograms***	170	2,683 (242)	18	625 (65)	3,039 (65)	10
Final resolution	7.8 Å	6.6 Å	8.3 Å	6.5 Å	5.6 Å	17.0 Å

Supplementary Table 1. Summary of cryo-electron tomography data acquisition and image processing.

* Icosahedral symmetry applied.

** Five- or six-fold symmetry applied.

*** In parentheses are the numbers of SLP or DLP particles included in sub-tomogram averaging of 5-fold and pseudo 6-fold maps.

	Ramachandran plot (%)			Bonds (RMSD)		Rotamer outliers (%)	Correlation coefficient	
Map and model	Favoured	Allowed	Outliers	Length (Å)	Angles(°)		Main chain	Side chain
SLP	86.40	13.50	0.10	0.998	0.006	0.40	0.79	0.77
DLP 5-fold	83.71	16.15	0.14	1.031	0.007	0.50	0.76	0.74
DLP pseudo 6-fold	90.00	9.93	0.07	0.921	0.006	0.31	0.78	0.77

Supplementary Table 2. Model refinement statistics.

Interface	Virion-like particle interface area (Å^2)	SLP interface area (Å^2)
A'B	5,900	4,400
AB	5,500	4,000
AB''	2,100	2,200
BB'	3,500	1,500
AA'	2,500	1,200
AA''	1,200	1,000

Supplementary Table 3. Changes in the interface areas between λ1 molecules.

References

1 Afonine, P. V. *et al.* New tools for the analysis and validation of cryo-EM maps and atomic models. *Acta Crystallographica Section D* **74**, 814-840, doi:doi:10.1107/S2059798318009324 (2018).

2 Williams, C. J. *et al.* MolProbity: More and better reference data for improved all-atom structure validation. *Protein Science* **27**, 293-315, doi:10.1002/pro.3330 (2018).

3 Xiao, C. A. & Rossmann, M. G. Interpretation of electron density with stereographic roadmap projections. *Journal of Structural Biology* **158**, 182-187, doi:10.1016/j.jsb.2006.10.013 (2007).
