# Supplementary material for: Assembly intermediates of orthoreovirus captured in the cell
Source: Nat Commun. 2020 Sep 7;11:4445. doi: 10.1038/s41467-020-18243-9 (PMC7477198; doi:10.1038/s41467-020-18243-9)
Supplement: Supplementary file 3 — Reporting Summary [file 41467_2020_18243_MOESM3_ESM.pdf]

nature research

Corresponding author(s): David Stuart

Last updated by author(s): 3 Aug 2020

Reporting Summary

Nature Research wishes to improve the reproducibility of the work that we publish. This form provides structure for consistency and transparency in reporting. For further information on Nature Research policies, see our Editorial Policies and the Editorial Policy Checklist.

Statistics

For all statistical analyses, confirm that the following items are present in the figure legend, table legend, main text, or Methods section.

n/a	Confirmed	
☐	☒	The exact sample size (*n*) for each experimental group/condition, given as a discrete number and unit of measurement
☒	☐	A statement on whether measurements were taken from distinct samples or whether the same sample was measured repeatedly
☒	☐	The statistical test(s) used AND whether they are one- or two-sided<br>*Only common tests should be described solely by name; describe more complex techniques in the Methods section.*
☒	☐	A description of all covariates tested
☒	☐	A description of any assumptions or corrections, such as tests of normality and adjustment for multiple comparisons
☒	☐	A full description of the statistical parameters including central tendency (e.g. means) or other basic estimates (e.g. regression coefficient) AND variation (e.g. standard deviation) or associated estimates of uncertainty (e.g. confidence intervals)
☒	☐	For null hypothesis testing, the test statistic (e.g. F, t, r) with confidence intervals, effect sizes, degrees of freedom and P value noted<br>*Give P values as exact values whenever suitable.*
☒	☐	For Bayesian analysis, information on the choice of priors and Markov chain Monte Carlo settings
☒	☐	For hierarchical and complex designs, identification of the appropriate level for tests and full reporting of outcomes
☒	☐	Estimates of effect sizes (e.g. Cohen's *d*, Pearson's *r*), indicating how they were calculated

Our web collection on statistics for biologists contains articles on many of the points above.

Software and code

Policy information about availability of computer code

Data collection	Serial EM version 3.5.9
Data analysis	EM Clarity version 1.1.9.2, Protomo version 1.1.4, Appion version 3.3, PHENIX 1.13, Coot 0.8.9.2, IMOD 4.9, roadmap 4.5, ccp4 7.0.078

For manuscripts utilizing custom algorithms or software that are central to the research but not yet described in published literature, software must be made available to editors and reviewers. We strongly encourage code deposition in a community repository (e.g. GitHub). See the Nature Research guidelines for submitting code & software for further information.

Data

Policy information about availability of data

All manuscripts must include a data availability statement. This statement should provide the following information, where applicable:

Reovirus sequence data have been deposited at the NCBI, accession codes MT614295-7. Cryo-EM density maps of SLP 5-fold and virion-like particle 5-fold and pseudo 6-fold have been deposited in EMDB under the accession codes EMD-22165, EMD-22166 and EMD-22164. The resulting atomic models have been deposited in the Protein Data Bank under the accession codes PDB-6ZTS, PDB-6ZTZ and PDB-6ZTY.
The following PDB entries were used in the study:
1ej6 [http://dx.doi.org/10.2210/pdb1ej6/pdb]
2cse [http://dx.doi.org/10.2210/pdb2CSE/pdb]
1jmu [http://dx.doi.org/10.2210/pdb1JMU/pdb]
3s6x [http://dx.doi.org/10.2210/pdb3S6X/pdb]

Field-specific reporting

Please select the one below that is the best fit for your research. If you are not sure, read the appropriate sections before making your selection.

☒ Life sciences ☐ Behavioural & social sciences ☐ Ecological, evolutionary & environmental sciences

For a reference copy of the document with all sections, see nature.com/documents/nr-reporting-summary-flat.pdf

Life sciences study design

All studies must disclose on these points even when the disclosure is negative.

Sample size	The number particles used for each analysis is recorded in the paper. Particles were selected by eye.
Data exclusions	All good tomograms and particles were used
Replication	N/A. All available data were used to maximise the accuracy
Randomization	N/A. For calculation of resolution correlation of half data sets was used.
Blinding	N/A. Not required the particles were identifiable unambiguously be computational methods.

Reporting for specific materials, systems and methods

We require information from authors about some types of materials, experimental systems and methods used in many studies. Here, indicate whether each material, system or method listed is relevant to your study. If you are not sure if a list item applies to your research, read the appropriate section before selecting a response.

Materials & experimental systems

n/a	Involved in the study	
☒	☐	Antibodies
☐	☒	Eukaryotic cell lines
☒	☐	Palaeontology and archaeology
☒	☐	Animals and other organisms
☒	☐	Human research participants
☒	☐	Clinical data
☒	☐	Dual use research of concern

Methods

n/a	Involved in the study	
☒	☐	ChIP-seq
☒	☐	Flow cytometry
☒	☐	MRI-based neuroimaging

Eukaryotic cell lines

Policy information about cell lines

Cell line source(s)	MA104 were a gift from Prof McCrae of Warwick University UK.
Authentication	Not authenticated by us.
Mycoplasma contamination	not tested
Commonly misidentified lines (See ICLAC register)	N/A
