# Supplementary material for: Assembly intermediates of orthoreovirus captured in the cell
Source: Nat Commun. 2020 Sep 7;11:4445. doi: 10.1038/s41467-020-18243-9 (PMC7477198; doi:10.1038/s41467-020-18243-9)
Supplement: Supplementary file 4 — Description of Additional Supplementary Files [file 41467_2020_18243_MOESM4_ESM.pdf]

Description of Additional Supplementary Files

File Name: Supplementary Movie 1
Description: Part of a tomogram from which some of the particles used were picked.

File Name: Supplementary Movie 2
Description: SLP emClarity reconstruction.

File Name: Supplementary Movie 3
Description: Empty virion-like particle emClarity reconstruction.

File Name: Supplementary Movie 4
Description: Morph the λ1 proteins of the SLP "expanding" to the virion-like particle.
